# Supplementary material for: Improved Immunogenicity of the Inactivated F Genotype Mumps Vaccine against Diverse Circulating Mumps Viruses in Mice
Source: Vaccines (Basel). 2023 Jan 1;11(1):106. doi: 10.3390/vaccines11010106 (PMC9862704; doi:10.3390/vaccines11010106)
Supplement: Supplementary file 1 [file vaccines-11-00106-s001.zip › vaccines-2054689-supplementary.pdf]

Supplementary Figure S1.

A

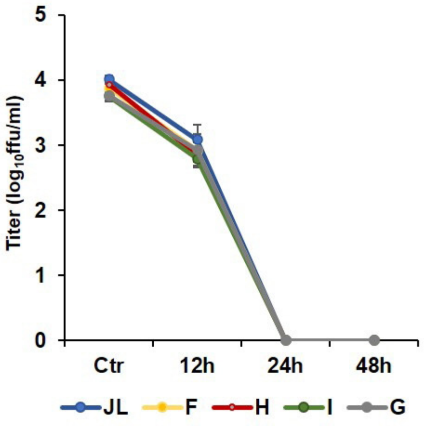

B

No.	Group name	Group		Dose
1	PBS	PBS		-
2	JL	Jeryl Lynn<br>A genotype	virus	2.5X105ffu
3	JL1		formalin inactivation	1μg
4	JL5			5μg
5	JL10			10μg
6	F	MuVi/Incheon.KOR/16.08/22[F]<br>F genotype	virus	2.5X105ffu
7	F1		formalin inactivation	1μg
8	F5			5μg
9	F10			10μg
10	H	MuVi/Gyeonggi-do.KOR/22.16/1[H]<br>H genotype	virus	2.5X105ffu
11	H1		formalin inactivation	1μg
12	H5			5μg
13	H10			10μg
14	I	MuVi/Jeonnam.KOR/10.15/5[I]<br>I genotype	virus	2.5X105ffu
15	I1		formalin inactivation	1μg
16	I5			5μg
17	I10			10μg
18	G	MuV/Iowa.US/2006[G]<br>G genotype	virus	2.5X105ffu
19	G1		formalin inactivation	1μg
20	G5			5μg
21	G10			10μg

Figure S1: Inactivation of MuV by incubation with formalin and humoral responses of inactivated mumps vaccine candidates. (A) Time-course of viral inactivation by formalin with JL (A genotype), F, H, I, and G genotypes of MuV were incubated with 0.05% formalin at 4 °C, and then the mixtures were harvested at various time points to measure viral titers using a focus-forming assay. (B) Ex-perimental groups and dosing schedule. BALB/c mice were immunized intramuscularly with 1, 5, and 10 μg of inactivated mumps vaccine candidates (JL, F, H, I, and G), 2.5 × 105 ffu/mL of MuV, which is the wild-type virus control, and Jeryl Lynn vaccine (genotype A) which is a positive control and a currently available vaccine at weeks 0 and 3. The blood samples were collected at week 6. The blue letters show the selected group for the main experiments.

Supplementary Figure S2.

A

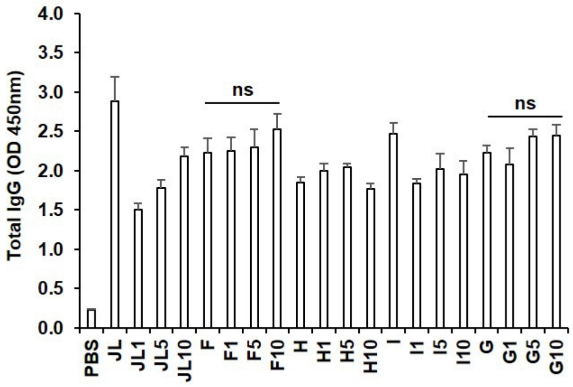

B

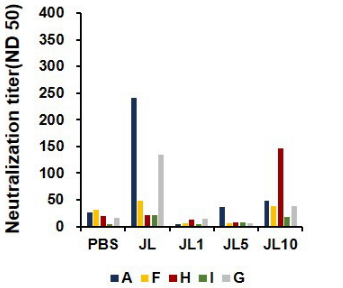

Group	Dose
PBS	-
JL	Jeryl Lynn [A] 2.5*10^5 ffu/ml
JL1	Inactivated JL [A] MuV 1 μg
JL5	Inactivated JL [A] MuV 5 μg
JL10	Inactivated JL [A] MuV 10 μg

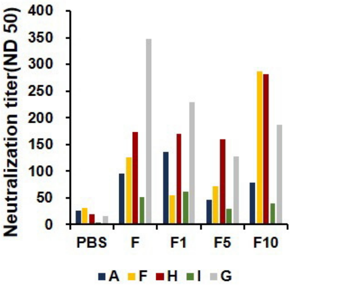

Group	Dose
PBS	-
F	MuVi/Incheon KOR/16.08/22[F] 2.5*10^5 ffu/ml
F1	Inactivated [F] MuV 1 μg
F5	Inactivated [F] MuV 5 μg
F10	Inactivated [F] MuV 10 μg

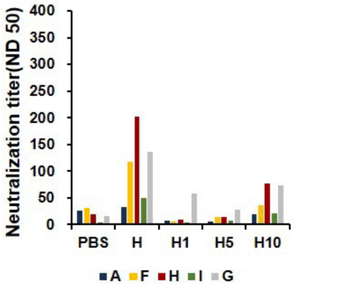

Group	Dose
PBS	-
H	MuVi/Gyeonggi KOR/26.16/1[H] 2.5*10^5 ffu/ml
H1	Inactivated [H] MuV 1 μg
H5	Inactivated [H] MuV 5 μg
H10	Inactivated [H] MuV 10 μg

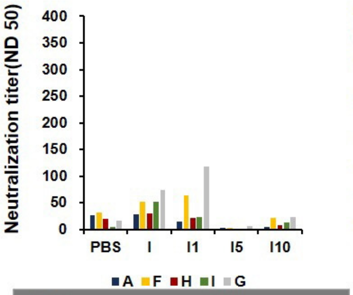

Group	Dose
PBS	-
I	MuVi/Jeonnam KOR/10.15/5[I] 2.5*10^5 ffu/ml
I1	Inactivated [I] MuV 1 μg
I5	Inactivated [I] MuV 5 μg
I10	Inactivated [I] MuV 10 μg

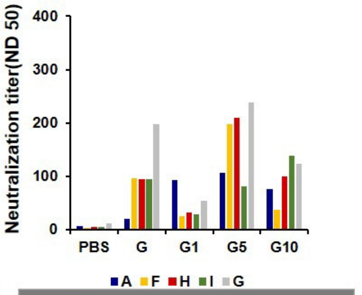

Group	Dose
PBS	-
G	MuV/Iowa.US/2006[G] 2.5*10^5 ffu/ml
G1	Inactivated [G] MuV 1 μg
G5	Inactivated [G] MuV 5 μg
G10	Inactivated [G] MuV 10 μg

Figure S2: Humoral responses of inactivated mumps vaccine candidates. (A) Total IgG elicited in BALB/c mice serum among dif-ferent immune groups measured by ELISA. All vaccinated mice induced a significant MuV-specific IgG response. Not significant (NS) when compared to JL group. (B) Serum neutralizing antibody responses against diverse genotypes of MuV at 6 weeks. The average number of spots was calcu-lated in triplicate. The results of 50% neutralizing antibody titers are presented as the mean values from pooled mouse serum (N = 3).

Supplementary Figure S3.

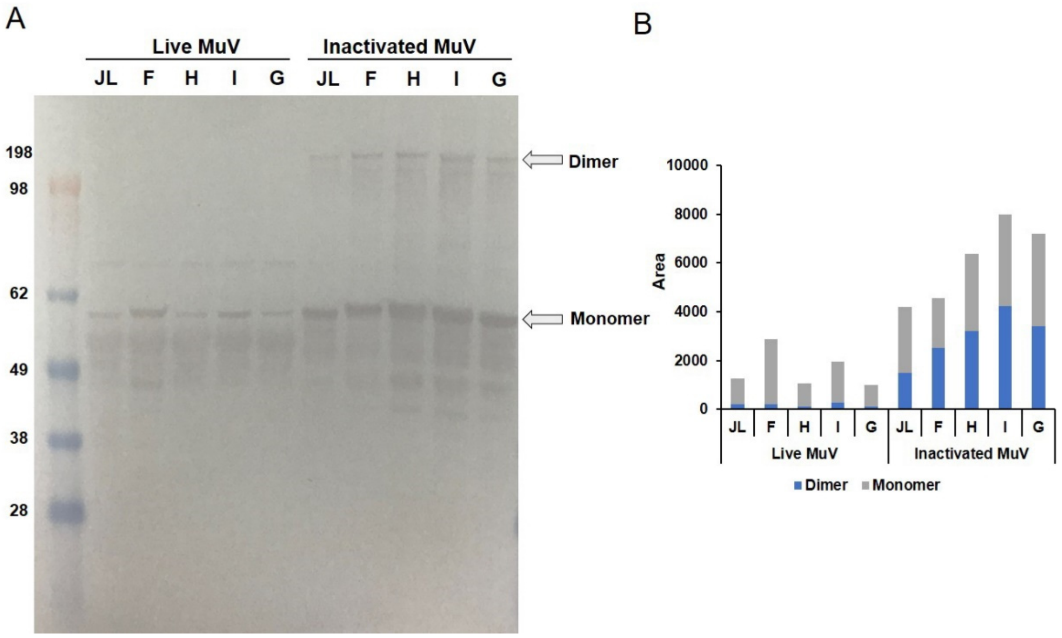

Figure S3: (A) Representative immunoblot images of purified inactivated MuV of the HN proteins. Lanes 1–5 are JL, F, H, I, and G genotypes in live virus cultured medium, and lanes 6–10 show the inactivated virions of MuV. (B) The graph shows the band in-tensity of the HN proteins as measured by ImageJ.

Supplementary Figure S4.

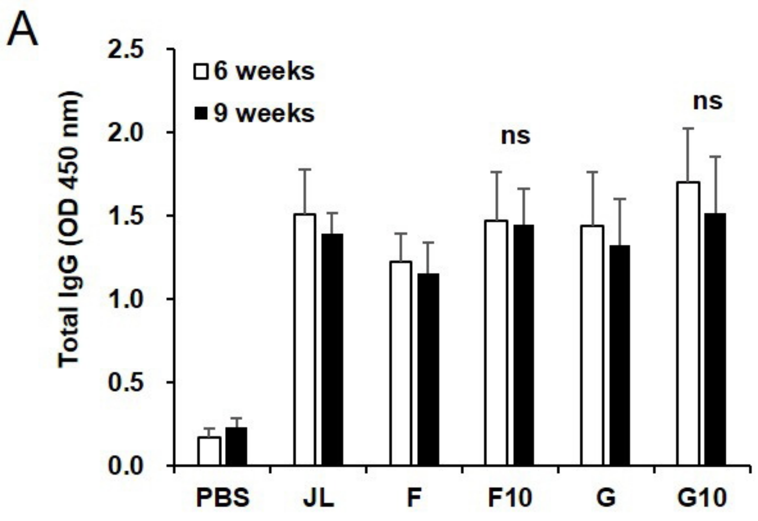

Figure S4: (A) Total IgG elicited by the inactivated vaccine candidates, measured by ELISA at 6 and 9 weeks. Not significant (NS) when compared to JL group.
